# Serial interval and incubation period of COVID-19: a systematic review and meta-analysis

**DOI:** 10.1186/s12879-021-05950-x

**Published:** 2021-03-11

**Authors:** Muluneh Alene, Leltework Yismaw, Moges Agazhe Assemie, Daniel Bekele Ketema, Wodaje Gietaneh, Tilahun Yemanu Birhan

**Affiliations:** 1 Department of Public Health, Debre Markos University, Debre Markos, Ethiopia; 2 Department of Epidemiology and Biostatistics, Institute of Public Health, College of Medicine and Health Science, University of Gondar, Gondar, Ethiopia

**Keywords:** COVID-19, Serial interval, Incubation period, Meta-analysis

## Abstract

**Background:**

Understanding the epidemiological parameters that determine the transmission dynamics of COVID-19 is essential for public health intervention. Globally, a number of studies were conducted to estimate the average serial interval and incubation period of COVID-19. Combining findings of existing studies that estimate the average serial interval and incubation period of COVID-19 significantly improves the quality of evidence. Hence, this study aimed to determine the overall average serial interval and incubation period of COVID-19.

**Methods:**

We followed the PRISMA checklist to present this study. A comprehensive search strategy was carried out from international electronic databases (Google Scholar, PubMed, Science Direct, Web of Science, CINAHL, and Cochrane Library) by two experienced reviewers (MAA and DBK) authors between the 1st of June and the 31st of July 2020. All observational studies either reporting the serial interval or incubation period in persons diagnosed with COVID-19 were included in this study. Heterogeneity across studies was assessed using the I^2^ and Higgins test. The NOS adapted for cross-sectional studies was used to evaluate the quality of studies. A random effect Meta-analysis was employed to determine the pooled estimate with 95% (CI). Microsoft Excel was used for data extraction and R software was used for analysis.

**Results:**

We combined a total of 23 studies to estimate the overall mean serial interval of COVID-19. The mean serial interval of COVID-19 ranged from 4. 2 to 7.5 days. Our meta-analysis showed that the weighted pooled mean serial interval of COVID-19 was 5.2 (95%CI: 4.9–5.5) days. Additionally, to pool the mean incubation period of COVID-19, we included 14 articles. The mean incubation period of COVID-19 also ranged from 4.8 to 9 days. Accordingly, the weighted pooled mean incubation period of COVID-19 was 6.5 (95%CI: 5.9–7.1) days.

**Conclusions:**

This systematic review and meta-analysis showed that the weighted pooled mean serial interval and incubation period of COVID-19 were 5.2, and 6.5 days, respectively. In this study, the average serial interval of COVID-19 is shorter than the average incubation period, which suggests that substantial numbers of COVID-19 cases will be attributed to presymptomatic transmission.

**Supplementary Information:**

The online version contains supplementary material available at 10.1186/s12879-021-05950-x.

## Background

The 2019 Coronavirus Disease (COVID-19) continues to be one of the potential clinical and public health issues in the global population [1]. Globally, from the outbreak of the virus up to August 5, 2020, 18 million total confirmed cases and 700, 000 deaths were reported [2]. Rapid spread of COVID-19 causes an enormous impact on social, economic and health care system in the world [3]. Effective treatment to block the spread of COVID-19 is not developed yet, hence countries implement non-treatment intervention such as social distancing, isolation, face mask and quarantine to reduce its rapid transmission [4, 5].

Existing evidence showed that most of the COVID-19 cases are missed by screening due to they are unaware they were exposed, and not developed symptoms yet [5]. In the absence of strong public health interventions, preliminary estimates showed that the basic reproduction number of Severe Acute Respiratory Syndrome Coronovirus-2 (SARS-CoV-2) ranged from 2.8 to 5.5 [6]. Serial interval and incubation period are the two main epidemiological parameters that determine the transmission dynamics of infectious diseases [7]. Serial interval is defined as the time from illness onset in the primary case to illness onset in the secondary case, while incubation period is the time from infection occurred to the onset of signs and symptoms.

Previous studies reported that the average serial interval of COVID-19 is shorter than the average incubation period, which suggests that a substantial proportion of presymptomatic transmission [8, 9]. This makes it difficult to trace contacts due to the rapid turnover of case generations. An observational study that aimed to provide the epidemiological parameters of COVID-19 using seven countries data revealed that the mean incubation period and serial interval were 7.44 days and 6.70 days, respectively [10]. A study that compares the incubation period of SARS-CoV-2, severe acute respiratory syndrome coronavirus (SARS-CoV), and middle east respiratory syndrome coronavirus (MERS-CoV) reported that no observable difference in the incubation was noted between them [11].

Globally, a number of studies were conducted to estimate the average serial interval and incubation period of COVID-19. However, the reported estimate of serial interval and incubation period in these fragmented studies vary depending on the number of study participants recruited, the type of design employed, the data collection period, and the country in which the study conducted. Combined findings of existing studies significantly strengthen the quality of evidence investigating the average estimate of serial interval and incubation period of COVID-19. Thus, this meta-analysis was aimed to determine the overall pooled mean serial interval and incubation period of COVID-19 using available evidences. The findings of this study are intended to improve policies and strategies for better prevention and control of COVID-19.

## Methods

### Source of information

We identified relevant studies through searching electronic databases and gray literatures. Additionally, we were searched from the reference lists of all the included studies to identify any other studies that may have been missed by our search strategy.

### Searching for studies

We followed the preferred reporting items for systematic review and meta-analysis (PRISMA) checklist for this study [12]. A comprehensive search strategy was performed from international electronic databases (Google Scholar, PubMed, Science Direct, Web of Science, CINAHL, and Cochrane Library) by two experienced review (MAA and DBK) authors between 1st of June and the 31st of July 2020. The following searching terms are used from the above databases: “serial interval” OR “generation time” AND “incubation period” OR “infectious period” AND “COVID-19” OR “SARS-CoV-2” OR “novel coronavirus”.

### Inclusion criteria

#### Design

All observational studies either reporting the serial interval or incubation period of COVID-19.

#### Study setting

Worldwide.

#### Population

All age group.

#### Publication status

All published and unpublished articles.

#### Language

Only studies reporting using the English language.

#### Publication date

Published from the 1st of January 2020 to the 30th of June, 2020.

### Exclusion criteria

Articles that were not fully accessed after at least two email contacts of the principal investigator were excluded. In addition, we excluded case reports, letters, and review articles.

### Study selection

The eligibility assessment was undertaken by two (WG and TYB) authors, independently. The disagreement between two reviewers were fixed by consensus.

### Outcome measures and data extraction

This study has two outcome variables. The first is the average estimate of serial interval. The serial interval is defined as the time from illness onset in the primary case to illness onset in the secondary case. It also measured from pairs of cases with a clear infector–infectee relationship. The second outcome variable is the average estimate of the incubation period. Incubation period is defined as the time from infection occurred to the onset of signs and symptoms. It was measured with cases of a well-defined period of exposure and symptom onset. Screening of studies and all essential data from the included studies were extracted independently by two (MA and LY) of the authors. This form includes the last name of the first author, country, data collection period, sample size, average estimate, standard deviation, and 95% confidence intervals. The same data extraction form was used for both outcomes. Discrepancies between the two reviewers was resolved by consensus involving all authors.

### Assessing the risk of bias

Two experienced reviewers (MA and DBK) were assessed the risk of bias of the included articles. The Newcastle-Ottawa Scale (NOS) adapted for cross-sectional studies was used to evaluate the quality of studies [13]. This tool includes three categories with a maximum score of 9 points. The first is the “selection” category, which accounts for a maximum of 4 points, the second is the “Comparability” category, which accounts for a maximum of 2 points, and the third is “outcome” which accounts a maximum of 3 points. Based on the composite score from this three categories, the studies were classified as good quality if the score ≥ 6 points, fair quality 2 to 5 points inclusively and poor quality ≤1 point.

### Data processing and analysis

A meta-analysis of continuous outcomes was employed for this study. We analyzed the data sets for each outcome variable (serial interval and incubation period). After extracting all essential data using Microsoft Excel, data were exported to R 4.0.2 statistical software for meta-analysis. In order to pool the results of included studies in a consistent format, we estimated the sample mean and standard deviation for studies that report median and interquartile range [14]. To determine the extent of variation between the studies, we did a heterogeneity test using the Higgins method, that was quantified by I^2^ value [15]. Weighted average using the inverse variance method was used to estimate the pooled average. A random-effect meta-analysis with an estimation of DerSimonian and Laird method was performed. The publication bias was also assessed using a funnel plot and Egger’s tests [16]. The pooled average estimates with 95%CI confidence interval was presented using forest plots.

## Results

### Search results

**Fig. 1:**
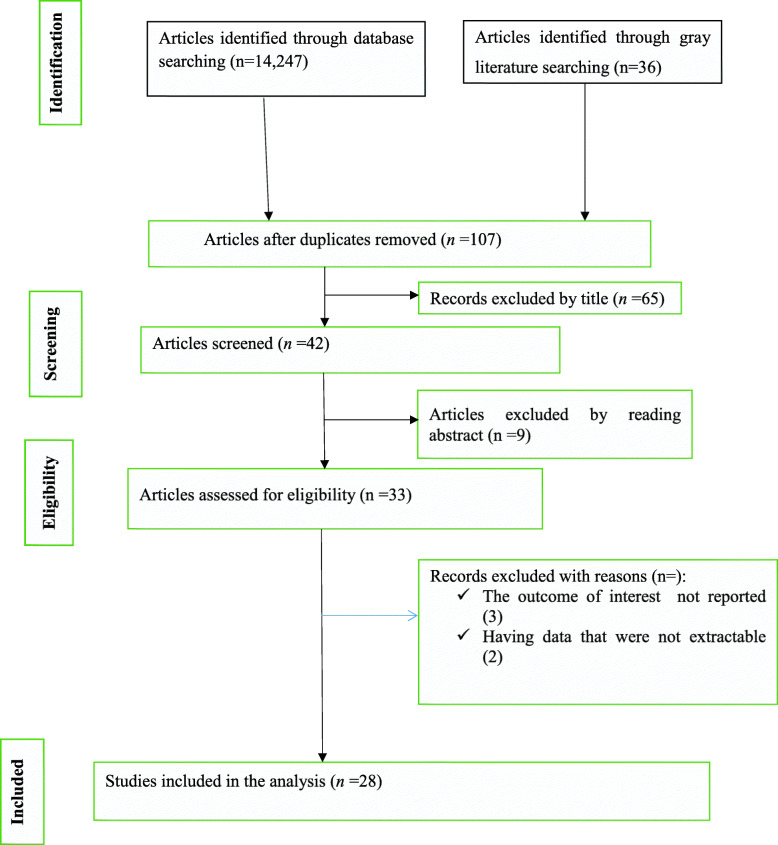
Flow chart diagram describing the selection of studies included in the meta-analysis of serial interval and incubation period of COVID-19, 2020

Figure 1 indicates the overall flow of study selection, literature search and number of the included studies. During electronic literature search 14,247 articles were identified and 14, 140 duplicated articles were removed. After meticulous review of the whole articles, 28 studies that fulfill the suitability standards were included. From the included studies, a single study might report both outcomes (serial interval and incubation period). Accordingly, a total of 23 and 14 studies were combined to estimate the mean serial interval, and incubation period of COVID-19, respectively.

### Description of the included studies

All the included studies are cross-sectional, and half of them were preprints. Majority of studies included in this study are conducted in China. We included a total of 23 articles to pool the mean of serial interval of COVID-19. The minimum and maximum pairs of COVID-19 patients among the included studies were 6 [17] and 1407 [18], respectively. Among the included studies, the mean serial interval of COVID-19 was ranged from 4. 2 days [19] to 7.5 days [17] (Table 1).

**Table 1:** Descriptions of the included studies conducted on the average estimate of serial interval of COVID-19, 2020

No.	First author	Country	Study period	Sample size (in pairs)	Mean in days	Standard deviation	95%CI for mean
1.	Aghaali et al	Iran	February 20,2020	37	4.55	3.3	NR
2.	Ali et al	China	January 9 to February 13, 2020	677	5.1	5.3	4.7–5.5
3.	Bi et al	China	Jan 14 to February 12, 2020	48	6·3	4.2	5.2–7·6
4.	Bui et al	Vietnam	January 29 to March 24,2020	9	5.8	3.6	NR
5	Cereda et al.	Italy	March 82,020	90	6.6	28	0.7–19
6.	Chan et al	China	January 23 to April 6, 2020	47	6.5	4.7	NR
7.	Cheng et al	Taiwan	January 15 to February 26,2020	12	7.0	5.8	3.7–13.2
8.	Du et al	China	January 20 to February 19, 2020	339	5.3	5.3	4.7–5.9
9.	He et al	China	January 21 to March 6, 2020	77	5.8	4.5	4.8–6.8
10.	Li et al	China	January 21, 2020, to February 29, 2020.	337	5.8	3.9	5:4–6:2
11.	Li et al	China	January 22, 2020	6	7.5	3.4	5.3–19
12.	Liu et al	China	January 1, to March 12, 2020	116	5.8	3.2	
13.	Najafi et al	Iran	February 22 to March 29, 2020	21	5.7	3.9	NR
14.	Nishiuraa et al	Japan	February 12, 2020	28	4.7	2.9	3.7–6.0
15.	Kowk et al	China	February 13,2020	26	4.6	3.3	3.4–5.9
16.	Tindale et al	Singapore	January 19 to February 26,2020	93	4.6	0.9	2.7–6.4
17.	Tindale et al	Tianjin	January 21 to February 27,2020	135	4.2	4.0	3.4–5.0
18.	Viego et al	Argentina	March 20 to May 8, 2020	13	5.5	5.0	2.8–8.1.
19.	Xu et al	China	January 15 to February 29, 2020	1407	5.2	5.3	4.6, 5.8
20.	Yang et al	China	January 20, 2020	152	4.6	4.4	3.7–5.5
21.	You et al	China	March 31, 2020	198	4.6	5.5	NR
22.	Zhang et al	China	after Jan. 20, 2020	35	5.1	3.4	1·3–11·6
23.	Zhao et al	China	February 15,2020	21	4.4	3	2.9–6.7

Similarly, to pool the mean incubation period of COVID-19, a total of 14 articles were included. Among those, the minimum sample size was 10 [17] and the maximum was 183 [20]. The mean incubation period of COVID-19 ranged from 4.8 days [20] to 9 days [19] (Table 2).

**Table 2:** Descriptions of the included studies conducted on the average incubation period of COVID-19, 2020

No.	First author	Country	Study period	Sample size	Mean in days	Standard deviation	95% CI
1.	Backer et al	China	January 20 to 28, 2020	88	6.4	3.8	5.6–7.7
2.	Bi et al	China	Jan 14 to Feb 12, 2020	183	4·8	0.9	4·2–5·4
3.	Cheng et al	Taiwan	January 15 to February 26,2020	32	4.9	6.3	2.7–8.4
4.	Han et al	China	December 29, 2019, to February 5, 2020.	59	5.8	2.9	5.1–6.5
5.	Kong	China	January 22 to February 15, 2020	136	8.5	4.1	7.8–9.2
6.	Lauer et al	China	January 4 to February 24, 2020.	181	5.1	0.97	4.5–5.8
7.	Li et al	China	January 22, 2020	10	5.2	1.9	4.1–7.0
8.	Linton et al	China	January 31, 2020	158	5.6	2.8	5.0–6.3
9.	Tindale et al	Singapore	January 19 to February 26,2020	93	7.1	4.9	6.1–8.3
10.	Tindale et al	Tianjin	January 21 to February 27,2020	135	9 .0	6.5	7.9–10.2
11.	Viego et al	Argentina	March 20 to May 8, 2020	12	7.5	5.9	4.1–10.9
12.	Yang et al	China	January 20, 2020	178	8.5	3.8	4.8–6.0
13.	You et al	China	March 31, 2020	139	8	4.8	NR
14.	Zhang et al	China	after Jan. 20, 2020	49	5.2	12.1	1·8–12·4

### Pooled average estimate of serial interval and incubation period

**Fig. 2:**
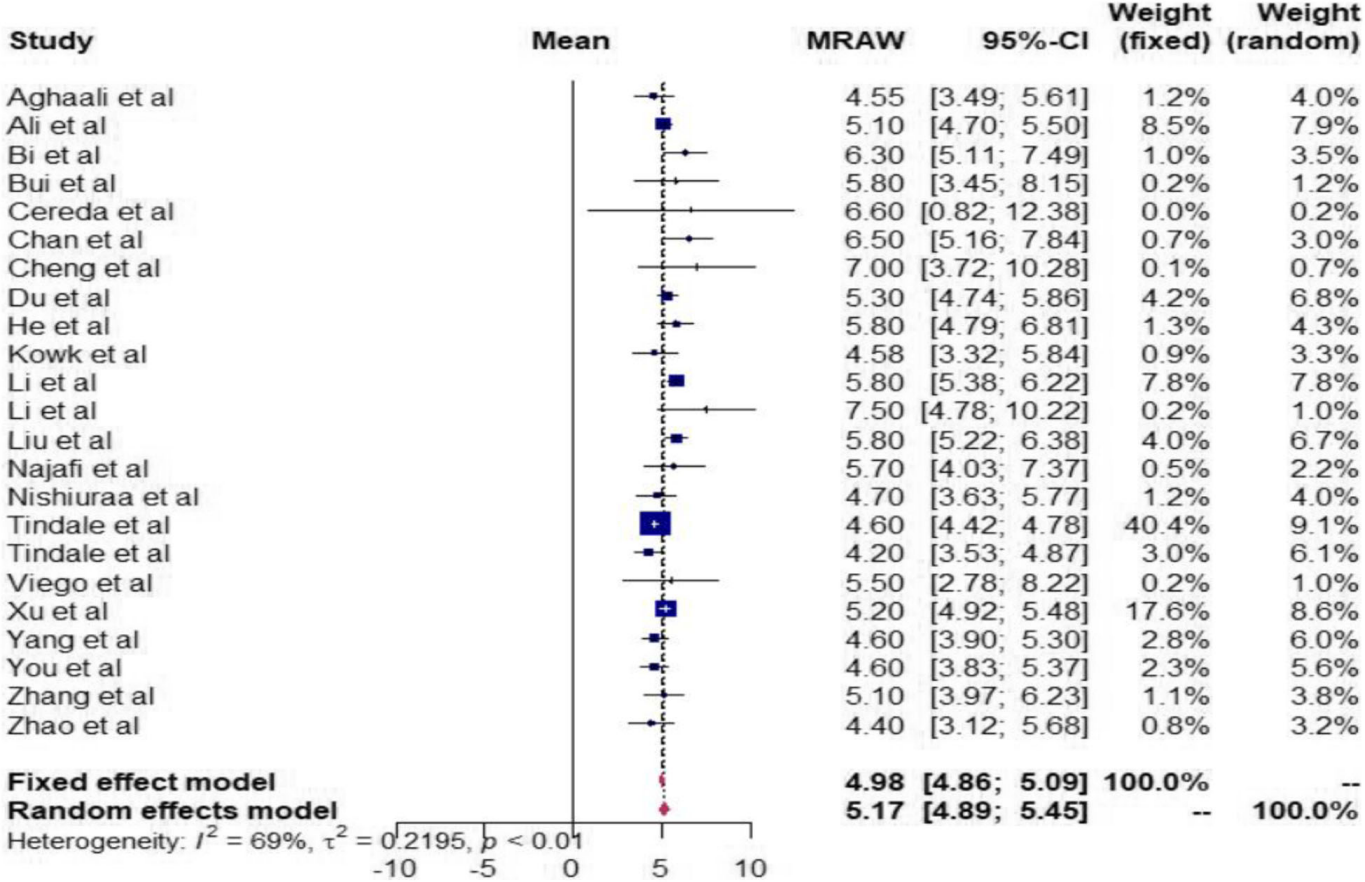
Forest plot that shows the pooled mean serial interval of COVID-19 using available studies, 2020

**Fig. 3:**
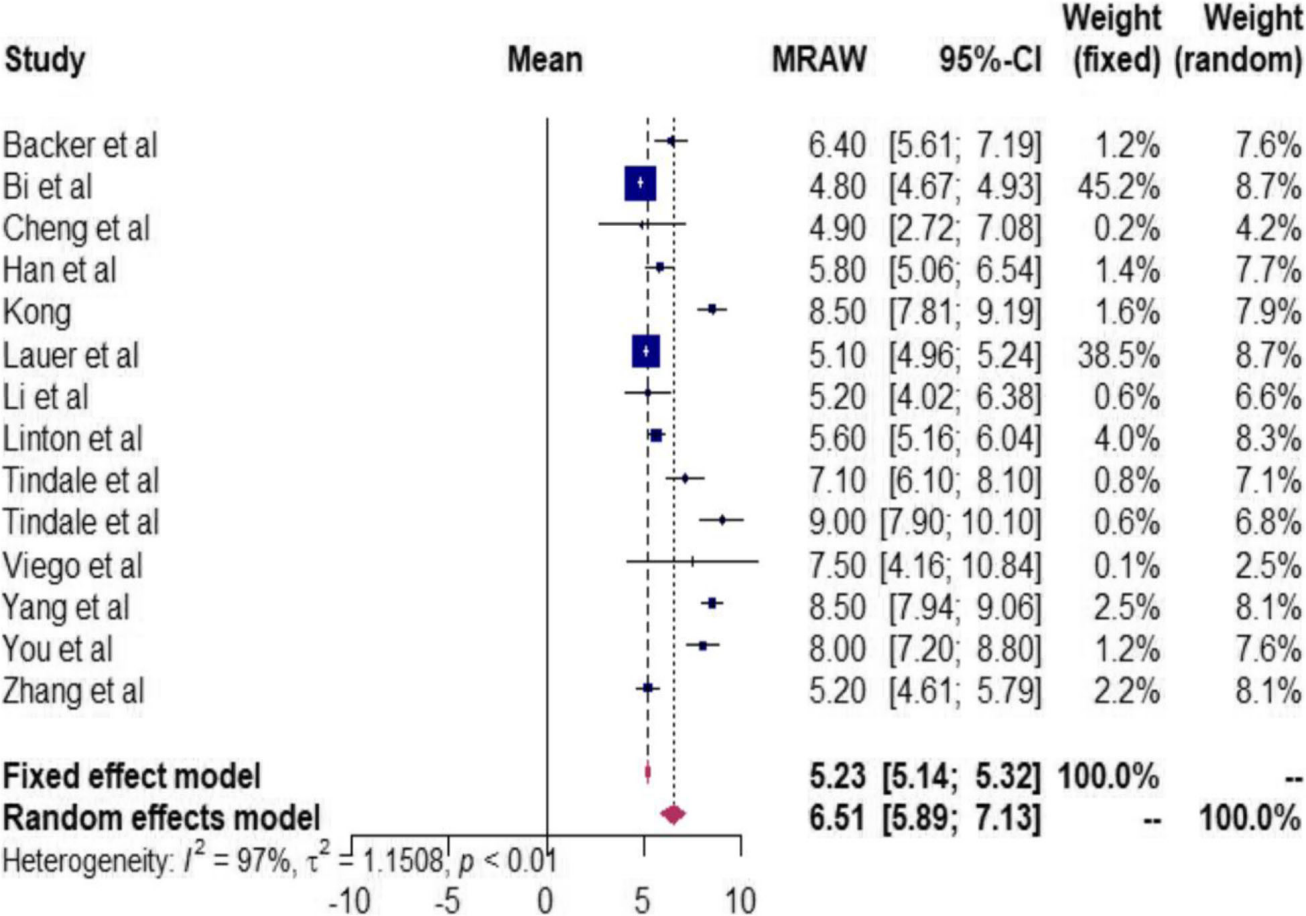
Forest plot that shows the pooled mean incubation period of COVID-19 using available studies, 2020

In this study, a total of 3924 pairs of COVID-19 patients were included to pool the mean serial interval. Accordingly, the weighted overall mean serial interval of COVID-19 was 5.2 (95%CI: 4.9–5.5) days (Fig. 2). Likewise, a total of 1, 453 COVID-19 patients were included to pool the overall incubation period of COVID-19. Consequently, the weighted pooled mean incubation period of COVID-19 was 6.5 (95%CI: 5.9–7.1) days (Fig. 3).

**Fig. 4:**
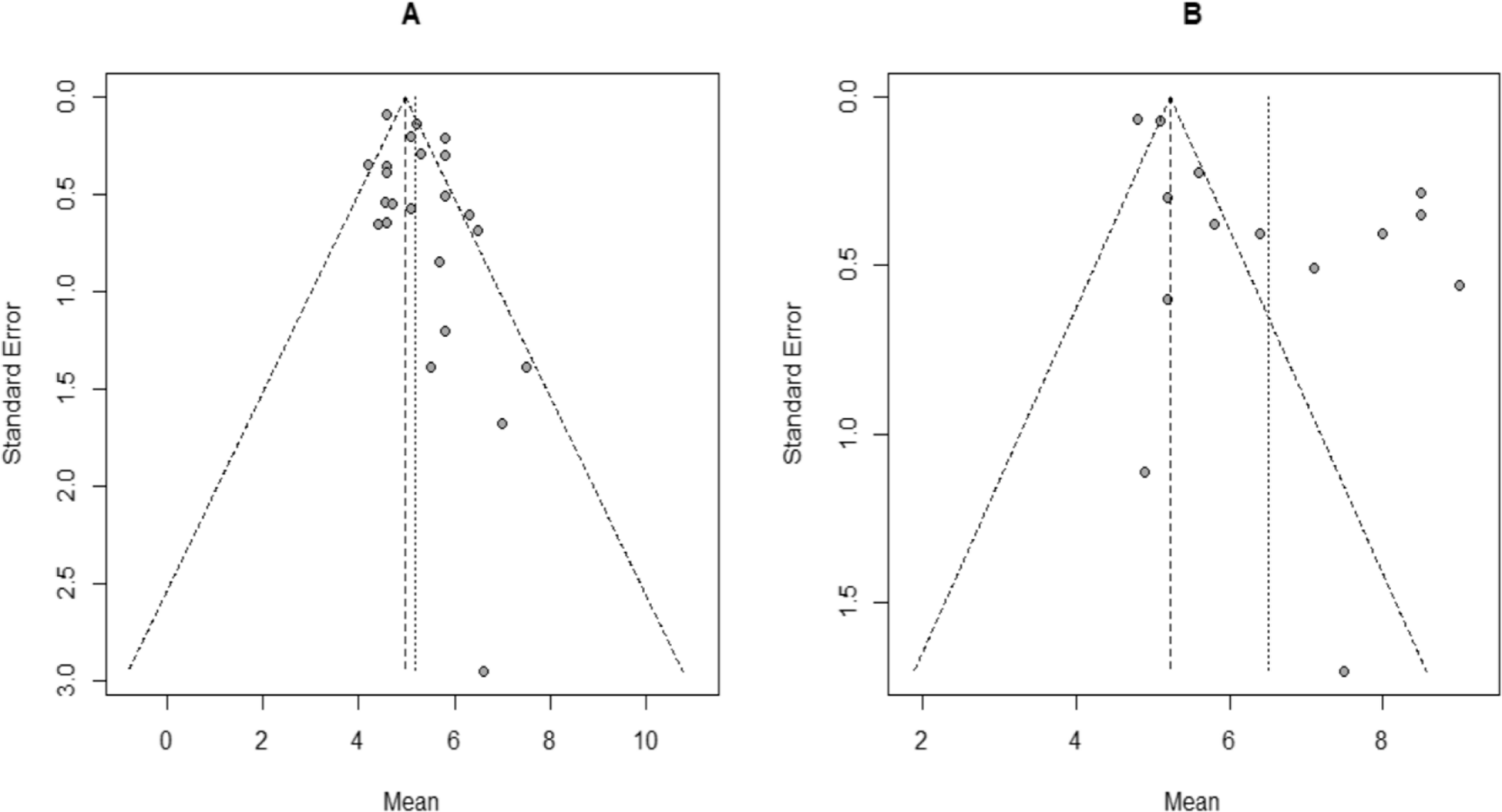
Funnel plots to check publication bias. **a** The included studies to pool the mean serial interval of COVID-19. **b** The included studies to pool the mean incubation period of COVID-19

Of the included studies to pool the mean serial interval of COVID-19, our summary quality assessment showed that nearly three-fourth (73.9%) of the studies had a good quality (Table S1). Similarly, among the included studies to pool the mean incubation period of COVID-19, about 71.4% of studies had a good quality (Table S2). We assessed the issue of publication bias by visual inspection of funnel plot and by using Egger’s regression test. Though the funnel plot looks asymmetrical the Egger’s test showed that no relationship between the effect size and its precision (Fig. 4).

## Discussion

The current study has two main objectives. The first objective is to determine the overall mean serial interval of COVID-19. In this study, we found that the weighted pooled mean serial interval of COVID-19 was 5.2 (95%CI: 4.9–5.5) days. This result is consistent with a study conducted in China [21], which reported that the mean serial interval of 5. 35 (95%CI: 4:63; 6:07) days. Another systematic review and meta-analysis study that combines 11 studies reported that the mean serial interval of 5.19 (95%CI: 4.37, 6.02) [22]. A study that compares the epidemiology of COVID-19, SARS-CoV, and MERS-CoV showed that COVID-19 had a short serial interval than SARS and MERS [23]. In addition, the pooled mean serial interval of COVID-19 obtained in this study is shorter than the mean serial interval of MERS and SARS reported in South Korea, and Singapore [24, 25].

The second objective of this study was to determine the overall mean incubation period of COVID-19. Consequently, the weighted pooled mean incubation period of COVID-19 was found 6.5 (95%CI: 5.9–7.1) days. This result is consistent with a study conducted in Hong Kong [26]. A result obtained from a rapid systematic review and meta-analysis showed that median incubation period of COVID-19 is 5.1 (95% CI: 4.5–5.8) days. Furthermore, the average incubation period of COVID-19 obtained in this study is longer than the average incubation period of SARS that reported in Toronto, Hong Kong, and Beijing [24, 27]. In addition, the average incubation period of COVID-19 obtained in the current study is longer than a systematic review study that reported the average incubation period of SARS [28].

Moreover, the average incubation period of COVID-19 obtained in the current study is longer than the mean incubation period of MERS reported in Hong Kong, and the Middle East [29, 30]. The possible explanation for this result might be the associations between shorter incubation periods and greater severity of infectious disease [31]. A longer incubation period was associated with a reduction in the risk of death [32]. The estimated fatality rate of COVID-19, SARS, and MERS are 2.3, 9.5, and 34.4%, respectively [33–35]. Conversely, another study showed that there is no observable difference between the incubation periods for SARS-CoV-2, severe acute respiratory syndrome coronavirus (SARS-CoV), and MERS-CoV. This study reported that the estimated incubation periods for SARS-CoV-2, SARS-CoV, and MERS-CoV were 4.9, 4.7, and 5.8 days, respectively [11].

In the current study, we included more studies by making longer searching date than the previous published articles. As the number of studies in meta-analysis increases, the power of estimating the pooled serial interval and incubation period of COVID-19 will be improved.

### Limitations

The current study has a number of limitations. Firstly, the overall estimate of serial interval and incubation period were computed with in a considerable heterogeneity. The source of heterogeneity might be difference in study population, data collection period, and method of analysis. Secondly, the majority of the included studies had relatively small study participants which may decrease the power of the study. Thirdly, the review was limited to only articles published in the English language. Lastly, since the included articles are limited to few countries, it may not represent the global figure.

## Conclusions

This systematic review and meta-analysis showed that the weighted pooled mean serial interval and incubation period of COVID-19 were 5.2, and 6.5 days, respectively. The average serial interval of COVID-19 is shorter than the average incubation period, which suggests that substantial numbers of COVID-19 cases will be attributed to presymptomatic or asymptomatic transmission.

## Supplementary Information

**Additional file 1: Table S1.** Assessing the risk of bias for the included studies to estimate the pooled avarage of serial interval of COVID-19, 2020.

**Additional file 2: Table S2.** Assessing the risk of bias for the included studies to estimate the pooled avarage incubation period of COVID-19, 2020.

## Data Availability

All the materials and data on which the findings of this review based are presented within the manuscript.

## Abbreviations

MERS-CoV: Middle East respiratory syndrome coronavirus
COVID-19: novel coronavirus disease 2019
SARS-CoV-2: Severe Acute Respiratory Syndrome Coronavirus 2
